# Impact of recurrence of hepatic cystic echinococcosis on postoperative outcomes in an endemic region of Chile: a retrospective cohort study

**DOI:** 10.1186/s41182-025-00832-3

**Published:** 2025-11-06

**Authors:** Josue Rivadeneira, Carlos Manterola, Luis Alvarado, Paola Simbaña-Garcia

**Affiliations:** 1 Doctorado en Ciencias Médicas, Universidad de La Frontera, Temuco, Chile; 2 Zero Biomedical Research, Quito, Ecuador; 3 Centro de Estudios Morfológicos y Quirúrgicos (CEMyQ), Universidad de La Frontera, Temuco, Chile; 4 Facultad de Medicina, Universidad Central del Ecuador, Quito, Ecuador

**Keywords:** “Postoperative Complications”[Mesh], “Mortality”[Mesh], “Length of Stay”[Mesh], “Recurrence”[Mesh], “Echinococcosis, Hepatic”[Mesh], “Cohort Studies”[Mesh], “Prognosis”[Mesh]

## Abstract

**Background:**

Hepatic cystic echinococcosis (HCE) remains a significant public health issue in endemic countries. Although recurrence is a recognized challenge, its independent impact on adverse clinical outcomes such as postoperative complications (POC), mortality, and length of hospital stay (LHS) remains poorly studied in Latin America. This study aimed to assess the risk of POC, mortality, and LHS in patients with recurrence of HCE.

**Methods:**

We conducted a retrospective cohort study of patients who underwent surgery for HCE between 1993 and 2019 at two centers in southern Chile. Patients with recurrence (exposed group) were compared to those undergoing primary surgery (non-exposed group). The primary outcome was the presence of POC; secondary outcomes included mortality and LHS. Crude and adjusted relative risks (RR) with 95% confidence intervals were estimated using Poisson regression with robust errors. Linear regression models were applied to assess the effect of recurrence on LHS.

**Results:**

A total of 154 patients with 271 cysts were included. Recurrence was identified in 43 patients (27.9%). POC occurred in 18.2% of the total cohort and were significantly more frequent in the recurrence group (41.9% vs. 9.0%, *p* < 0.001). Adjusted RR for POC in the presence of recurrence was 5.1 (95% CI 2.7–9.9). Mortality was higher in patients with recurrence (7.0% vs. 2.7%, RR: 2.6; 95% CI 0.5–12.3), though not statistically significant. LHS was 1 day longer in the recurrence group (7.3 ± 4.5 vs. 5.6 ± 3.4; *p* = 0.02), but this association lost significance in regression models.

**Conclusions:**

Recurrence of HCE increases the risk of POC. While trends toward higher mortality and prolonged LHS were observed, these did not reach statistical significance. These findings underscore the importance of long-term follow-up and the need to identify prognostic factors for recurrence to optimize outcomes in patients with HCE in endemic regions.

**Graphical abstract:**

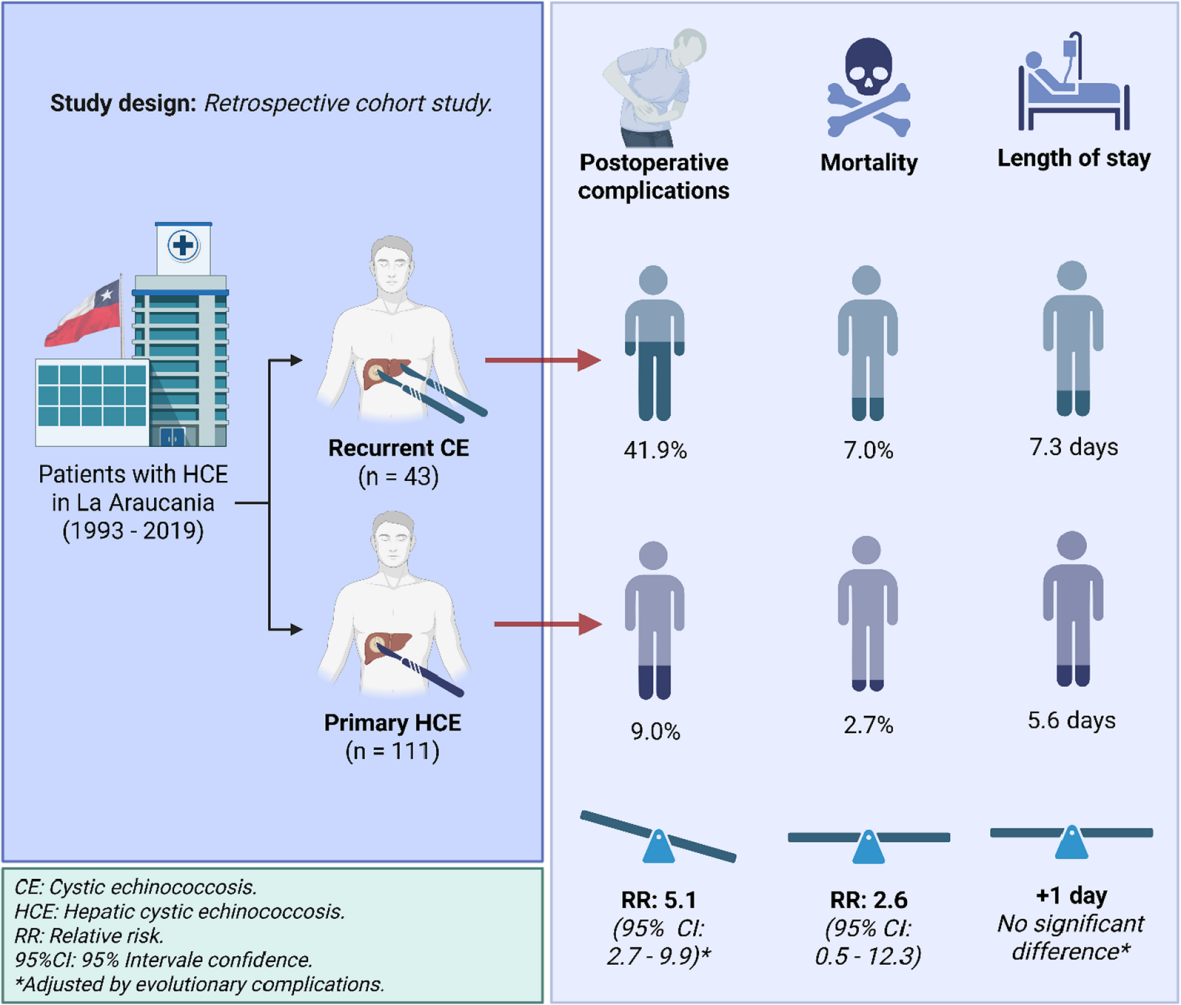

## Background

Cystic echinococcosis (CE), caused by the metacestode of *Echinococcus granulosus*, is one of the parasitic zoonoses with the greatest burden on human and animal health worldwide [1, 2]. According to the World Health Organization (WHO), CE has a global distribution, with incidence rates reaching up to 50 cases per 100,000 inhabitants in endemic regions of Africa, East Asia, the Middle East, and South America [3]. In Chile, an endemic country, the estimated national rate is 2 cases per 100,000 inhabitants, although in regions such as La Araucanía, rates increase up to 6 per 100,000 inhabitants [4]. These figures underscore the persistence of CE as a public health problem in the country despite its lower average incidence compared with other endemic areas worldwide.

CE is considered one of the 20 neglected tropical diseases according to the WHO and, as of 2021, was responsible for 0.58 disability-adjusted life years (DALYs) per 100,000 population, imposing a significant disease burden, particularly in low-income countries [5]. In humans, accidental intermediate hosts of the parasite, hepatic involvement is the most common (70–80%), characterized by a chronic, silent, and progressive course that is often diagnosed incidentally [6, 7]. Despite the availability of various therapeutic options and the inherent risks (medical, percutaneous or surgical treatments), surgery remains the most frequently performed treatment, particularly in complicated or recurrent cases[8, 9].

One of the primary challenges in managing CE is postoperative recurrence, defined as the appearance of new active cysts after surgical intervention. Its estimated incidence is 8% (95% confidence interval [95% CI] 6–10%) [10], and has been associated with factors, such as the presence of multiple cysts [11, 12], larger cyst diameter [11, 13], evolutionary complications [12, 13], and the type of surgical procedure performed [14–16]. In addition, perioperative benzimidazole therapy is consistently recommended in clinical guidelines as a preventive measure; however, the certainty of evidence remains low and consistent effect estimates are lacking[6, 9, 17].

Patients undergoing reoperation for recurrent CE experience higher rates of postoperative complications (POC) (25.0% vs. 5.8%) and length of hospital stay ([LHS] 16.4 vs. 9.5 days), compared to those undergoing primary hepatic CE (HCE) surgery [18]. These differences significantly impact healthcare systems, increasing costs and negatively affecting patients’ quality of life [19, 20].

Despite these findings, it remains unclear whether recurrence independently increases the risk of POC, mortality, or LHS. Evidence on this topic remains scarce worldwide, with few studies specifically addressing the independent prognostic role of recurrence. Addressing this gap is essential to guide surgical decision-making, optimize resource allocation, and improve patient outcomes in endemic and resource-limited settings.

This study aimed to assess the risk of POC, mortality, and LHS in patients with recurrence of HCE.

## Materials and methods

This manuscript adheres to the STROBE (Strengthening the Reporting of Observational Studies in Epidemiology) guidelines [21].

### Study design

Retrospective cohort study.

### Setting and participants

We included patients who underwent surgery for HCE between 1993 and 2019 at two centers in Temuco, Chile. Eligible patients were over 18 years, diagnosed with HCE via abdominal ultrasound (AU), and surgically treated.

The exposed group consisted of patients with recurrent EC, confirmed by AU. Recurrence was defined as the appearance of new active intra-abdominal cysts following surgical intervention for HCE [22]. The comparison group included patients undergoing their first surgical treatment for HCE, with at least 60 months of follow-up and no evidence of recurrence.

### Variables

The primary outcome was the occurrence of POC, defined as any deviation from the normal postoperative course within 30 days after surgery, according to the Clavien–Dindo classification. This definition included both surgical complications (e.g., infected biloma, biliary leak, surgical site infection, and abscess) and medical complications (e.g., pulmonary, gastrointestinal, cardiac, or urinary tract events) [23]. Secondary outcomes included POC severity (Clavien–Dindo classification), mortality (Yes/No), and LHS (number of days).

The independent variable was recurrence status. Confounding variables included age, comorbidities, number of cysts (single/multiple), maximum cyst diameter and location, presence of evolutionary complications (cyst infection, thoracic migration, cyst rupture into the biliary tract or peritoneum, cholangiohydatidosis, and anaphylaxis), WHO ultrasound classification (CE1, CE2, CE3 (a/b) y CE4) [9], and the type of initial surgery (conservative/radical) [24, 25]. Radical surgery was defined as total cystectomy and hepatic resections (anatomic or atypical). Surgical procedures were additionally classified according to international terminology into closed-cyst (total cystectomy and hepatic resections) and open-cyst (subtotal cystectomy) [8]. In this cohort, all radical procedures corresponded to closed-cyst surgeries, and all conservative procedures corresponded to open-cyst surgeries.

### Data collection

We used an anonymized institutional database with complete records for all included variables.

### Sample size

Estimated using the formula for comparing proportions in infinite populations, considering a 95% CI, 80% statistical power, and expected POC rates of 25.0% (recurrence CE) vs. 5.8% (primary HCE) [18]. A minimum of 43 patients per group was required. We employed census sampling, including all patients who met the inclusion criteria, thus minimizing selection bias.

### Statistical analysis

An exploratory analysis of the data was performed. Descriptive statistics were presented as frequencies, means or medians, and standard deviations (SD) or interquartile ranges. Inferential statistics were applied using Pearson's chi-squared or Fisher’s exact test for categorical variables, and Student’s *T* test or Mann–Whitney *U* test for continuous variables, according to distribution.

Crude relative risks (RR) with 95% CI were estimated for POC and mortality. Adjusted RR, were obtained using Poisson regression models with robust standard errors, controlling for covariates selected based on clinical relevance, following the rule of one independent variable per ten outcome events. We assessed overdispersion using the deviance/residual degrees of freedom ratio (> 1), and multicollinearity using the variance inflation factor (VIF, > 10). Model fit was evaluated using the Akaike Information Criterion (AIC), selecting the best-fitting model compared to the univariable model including only recurrence.

To assess the independent effect of recurrence on LHS, a robust multiple linear regression model was applied, using the MM method (M-estimators for scale and location). Covariates were selected manually based on clinical criteria. Model performance was assessed with adjusted *R*^2^ and robust residual errors (RRE), choosing the best-fitting model compared to the univariable model.

All analyses were performed with R software version 4.4.2 at a significance level of 0.05.

### Ethical considerations

The study was conducted in accordance with the Declaration of Helsinki and received approval from the RedSalud Scientific Ethics Committee (Code P-31.2024).

## Results

**Fig. 1:**
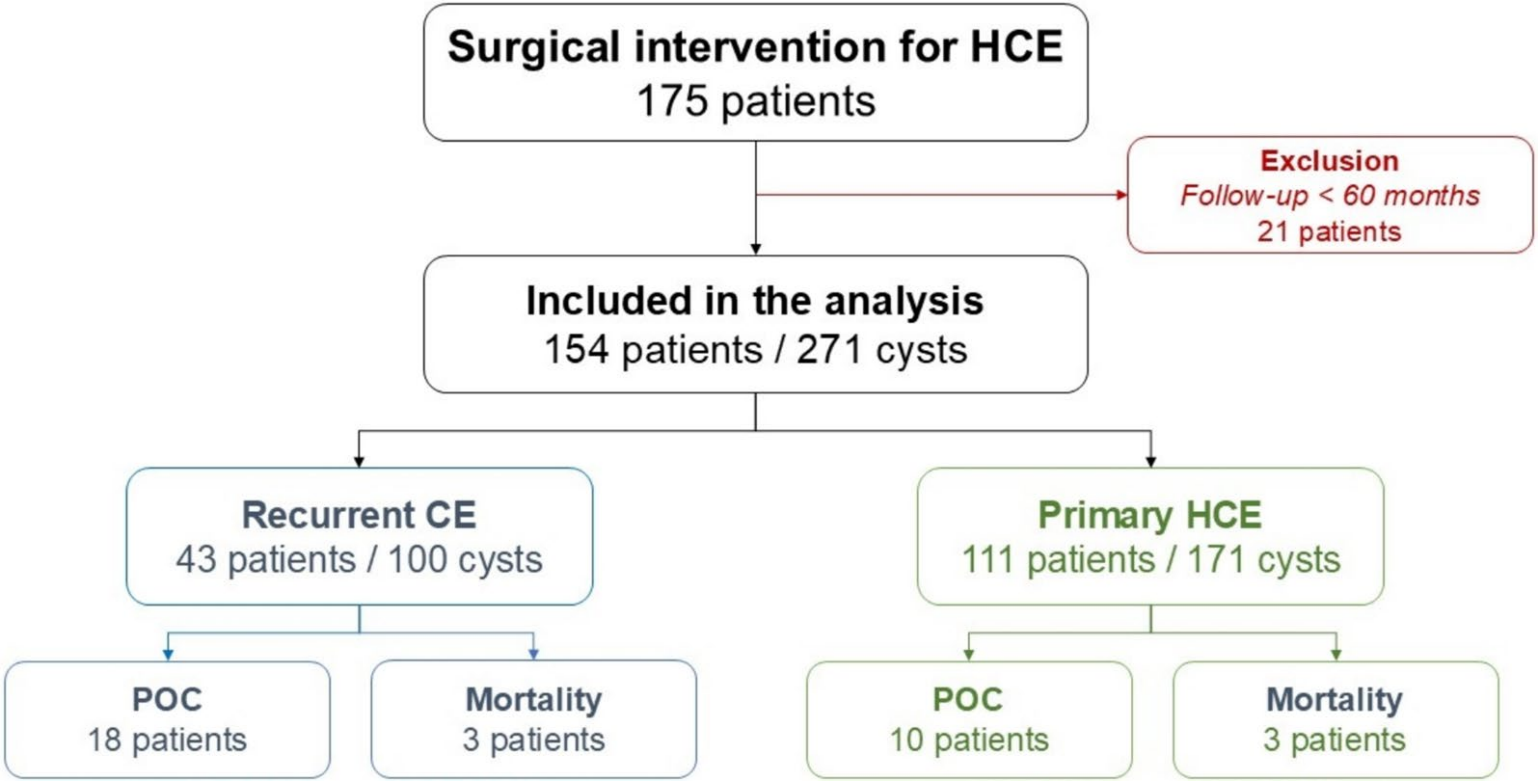
Participant flowchart. HCE: hepatic cyst echinococcosis; CE: cyst echinococcosis; POC: postoperative complications

During the study period, 175 patients undergoing surgical intervention for HCE were identified. After applying the selection criteria, 154 patients with 271 hepatic cysts were included in the analysis. Of these, 43 were diagnosed with recurrent EC (exposed group), and 111 comprised the non-exposed cohort (Fig. 1). No missing data were recorded, and all patients completed the follow-up period.

The mean age of the participants was 43 0.0 (SD = 15.5) years, and 55.8% were male. Comorbidities were present in 40.9% of patients, most commonly arterial hypertension (13.6%), cholelithiasis (11.0%), and heart disease (4.5%). There were no significant differences in sociodemographic or clinical characteristics between the two groups, except for a history of hypertension and liver disease (Tables 1 and 2).

**Table 1:** Sociodemographic, clinical, and surgical characteristics of patients undergoing surgery for HCE

	Total (*n* = 154)	Recurrent CE (*n* = 43)	Primary HCE (*n* = 111)	*p* value
Sex [*n* (%)]				
Male	86 (55.8)	24 (55.8)	62 (55.9)	0.99*
Comorbidities [*n* (%)]				
Presence of comorbidities	63 (40.9)	22 (51.2)	41 (36.9)	0.11*
Arterial hypertension	21 (13.6)	11 (25.6)	10 (9.0)	0.01*
Cholelithiasis	17 (11.0)	3 (7.0)	14 (12.6)	0.32**
Heart disease	7 (4.5)	2 (4.7)	5 (4.5)	0.97**
Liver disease	6 (3.9)	4 (9.3)	2 (1.8)	0.03**
Pulmonary disease	6 (3.9)	1 (2.3)	5 (4.5)	0.53**
Other	8 (5.2)	1 (2.3)	7 (6.3)	0.32**
Number of cysts [*n* (%)]				
Single	98 (63.6)	19 (44.2)	79 (71.2)	0.21*
Multiple (≥ 2)	56 (36.4)	24 (55.8)	32 (28.8)	
Location [*n* (%)]				< 0.001*
Right lobe	92 (59.7)	23 (53.5)	69 (62.2)	
Left lobe	31 (20.1)	4 (9.3)	27 (24.3)	
Bilateral	25 (16.2)	15 (34.9)	10 (9.0)	
Central hepatic	6 (3.9)	1 (2.3)	5 (4.5)	
Evolutionary complications [*n* (%)]				
Presence of complications	112 (72.7)	28 (65.1)	84 (75.7)	0.19*
Biliary communication	102 (66.2)	23 (53.5)	79 (71.2)	0.04*
Hepatic abscess	34 (22.1)	8 (18.6)	26 (23.4)	0.52*
Cholangiohydatidosis	11 (7.1)	4 (9.3)	7 (6.3)	0.52**
Thoracic migration	6 (3.9)	2 (4.7)	4 (3.6)	0.76**
Peritonitis	4 (2.6)	1 (2.3)	3 (2.7)	0.90**
Anaphylaxis	2 (1.3)	1 (2.3)	1 (0.9)	0.48**
WHO ultrasound classification [*n* (%)]				
CE1	70 (45.5)	19 (44.2)	51 (45.9)	0.63**
CE2	51 (33.1)	12 (27.9)	39 (35.1)	
CE3 (a/b)	27 (17.5)	10 (23.3)	17 (15.3)	
CE4	6 (3.9)	2 (4.7)	4 (3.6)	
Type of surgery [*n* (%)]				
Radical	134 (87.0)	34 (79.1)	100 (90.1)	0.07*
Conservative	20 (13.0)	9 (20.9)	11 (9.9)	
Type of surgery according to international terminology [*n* (%)]				
Open cyst	20 (13.0)	9 (20.9)	11 (9.9)	0.07*
Non-open cyst	134 (87.0)	34 (79.1)	100 (90.1)	

Bold = Statistically significant difference (*p* 0.05)

CE: Cystic echinococcosis

HCE: Hepatic cystic echinococcosis

*Chi-square test

**Fisher’s exact test

**Table 2:** Clinical and parasitological characteristics of patients undergoing surgery for HCE

	Total (*n* = 154)	Recurrent CE (*n* = 43)	Primary HCE (*n* = 111)	*p* value
Age (years)*	43 ± 15.5 (18–80)	46.3 ± 17.2 (20–80)	41.8 ± 14.7 (18–75)	0.11^†^
Number of cysts*	1.8 ± 1.5 (1–9)	2.3 ± 2.9 (1–8)	1.5 ± 1.1 (1–9)	**0.002**^**††**^
Largest cyst diameter (cm)*	15.1 ± 6.7 (5.0–30.0)	14.9 ± 6.8 (5.0–30.0)	15.3 ± 6.6 (5.0–30.0)	0.75^†^
Follow-up time (months)*	112.3 ± 36.4 (1–189)	105.9 ± 46.4 (1–186)	114.8 ± 31.6 (60–189)	0.47^†^

Bold = Statistically significant difference (*p* 0.05)

CE: Cystic echinococcosis

HCE: Hepatic cystic echinococcosis

*Mean ± standard deviation (min–max)

^†^Student’s *t* test

^††^Mann–Whitney *U* test

Multiple cysts were present in 36.6% of patients, with a mean maximum cyst diameter of 15 cm (range 5–30 cm), with no significant intergroup differences (Table 2). The majority of cysts (61.7%) were located in the right hepatic lobe, with a statistically significant difference between groups (*p* = 0.027). According to the WHO ultrasound classification, the most frequent stages were CE1 (45.5%) and CE2 (33.1%), with no significant intergroup differences (*p* = 0.63). Radical and non-open cyst surgical procedures were performed in 87.0% of patients, without statistically significant differences between the exposed and non-exposed groups (*p* = 0.06). Evolutionary complications of HCE were observed in 72.7% of patients, with no intergroup differences, except for biliary communication (*p* = 0.04) (Tables 1 and 2).

The median follow-up time in the recurrence group was 115 months (range 1–186), not significantly different from that of the non-exposed group (*p* = 0.46) (Table 2).

*Postoperative complications:* Overall, 18.2% of patients developed at least one POC, and 3.8% experienced severe events requiring surgical reintervention or intensive care (Clavien–Dindo ≥ III) (Table 3). Surgical site infections were the most common complications (39.3%), followed by pulmonary (25.0%) and gastrointestinal events (25.0%). Cardiac complications, urinary tract infections, and incisional hernias each accounted for 3.4%.

**Table 3:** Postoperative complications and mortality associated with CE recurrence

	Total (*n* = 154)	Recurrent CE (*n* = 43)	Primary HCE (*n* = 111)	*p* value	Crude RR (95% CI)
Postoperative complications [*n* (%)]	28 (18.2)	18 (41.9)	10 (9.0)	< 0.001*	4.7 (2.3–9.3)
Clavien–Dindo [*n* (%)]				0.66*	–
Grade I	14 (50.0)	10 (55.6)	4 (40.0)		
Grade II	8 (28.6)	4 (22.2)	4 (40.0)		
Grade ≥ IIIa	6 (21.5)	4 (22.2)	2 (20.0)		
Mortality [*n* (%)]	6 (3.9)	3 (7.0)	3 (2.7)	0.35**	2.6 (0.5–12.3)

RR: Relative risk

95% CI 95% Confidence Interval

^*^Chi-square test

^**^Fisher’s exact test

POC were significantly more common in the recurrence group compared to the non-exposed group (41.9% vs. 9.0%; *p* < 0.001), estimating a crude RR of 4.7 (95% CI 2.3–9.3) and an AIC of 139.5 (Table 3).

After adjusting for evolutionary complications in a multivariable model, the RR for recurrence and POC increased to 5.1 (95% CI 2.7–9.9). Evolutionary complications were independently associated with POC (RR: 3.8; 95% CI 1.3–10.9). The adjusted model demonstrated a better fit than the univariable model (AIC: 134.9) (Table 4).

**Table 4:** Postoperative complications, Poisson regression with robust coefficients

Variable	Coefficient	RR (95% CI)	*p* value
Intercept	− 3.6	0.03 (0.01–0.1)	< 0.001
Recurrent CE vs. primary HCE	1.6	5.1 (2.7–9.9)	< 0.001
Evolutionary complications vs. no complications	1.3	3.8 (1.3–10.9)	0.01
Deviance: 72.85/Residual degrees of freedom: 151/VIF: 1.00/AIC: 134.85			

CE: Cystic echinococcosis

HCE: Hepatic cystic echinococcosis

RR: Relative Risk

95% CI 95% Confidence Interval

*Mortality:* The mortality rate was 3.9% with 7.0% in the exposed group versus 2.7% in the non-exposed group (RR: 2.6; 95% CI 0.5–12.3; *p* = 0.35) (Table 3). Due to the low event count, multivariable analysis was not performed for this outcome.

*Length of stay:* LHS average was 6 ± 4 days. A statistically significant difference of 1 day (95% CI 0.0004–2.0; *p* = 0.02) was observed, with longer stays among patients in the recurrence group (7.3 ± 4.5 vs. 5.6 ± days).

In the univariable robust linear regression, recurrence was associated with an additional 0.6 days of hospitalization (*β* = 0.57; 95% CI [− 0.4]–0.5; *p* = 0.24). After adjusting for evolutionary complications, the difference slightly increased but remained non-significant (*β* = 0.64; 95% CI [− 0.3]–1.6; *p* = 0.17) (Table 5). Both models identified 11 outliers. The multivariable model explained slightly more variability (adjusted *R*^2^: 0.018 vs. 0.013) and maintained the same relative root error (RRE: 1.7).

**Table 5:** Length of hospital stay, robust linear regression analysis (MM method)

	Coefficient	95% CI	*p* value
Intercept	4.4	3.7–5.2	** < 0.001**
Recurrent CE vs. primary HCE	0.6	(− 0.3)–1.6	0.17
Evolutionary complications vs. no complications	0.4	(− 0.4)–1.2	0.30
Adjusted *R*^2^: 0,02/Robust residual errors: 1,74			

Bold = Statistically significant difference (*p* 0.05)

CE: Cystic echinococcosis

HCE: Hepatic cystic echinococcosis

95% CI 95% Confidence Interval

## Discussion

Recurrence remains one of the most significant challenges in the surgical treatment of HCE [6, 16]. Despite its clinical relevance, evidence regarding its effect on postoperative outcomes such as POC, mortality, and LHS remains limited, particularly in Latin American countries, where the disease burden is still considerable [5, 26]. This is one of the few studies in which recurrence is evaluated as an independent risk factor for these outcomes. It was conducted with homogeneous clinical characteristics and long-term follow-up, representing a type 2b level of evidence for prognosis studies, according to the Oxford Center for Evidence-Based Medicine.

In our sample, 18.2% of patients presented at least one POC, with a 32.9% absolute increase in the recurrence group (41.9% vs. 9.0%). These results align with previous findings that describe higher complication rates in patients undergoing surgery for recurrent CE [18, 27, 28]. After adjusting for evolutionary complications, which are recognized as a predisposing factor for adverse postoperative events [29, 30], the association remained significant (RR: 5.1; 95% CI 2.7–9.9), confirming recurrence as an independent risk factor. A previous Chilean study reported a similar adjusted odds ratio of 4.1 (95% CI 1.3–13.2) for recurrence and POC [28].

This association is likely attributable to increased surgical complexity in recurrent cases. Adhesions, fibrosis, anatomical distortion, and tissue fragility resulting from prior surgeries contribute to higher intraoperative risks, including bile duct injury, hemorrhage, abscess formation, and wound infections [18].

The mortality rate in this study was low (3.9%), consistent with literature reports indicating mortality rates between 0.8% and 5.2%, depending on cyst characteristics and surgical approach [26]. We observed an absolute difference of 4.3%, although not statistically significant (RR: 2.6; 95% CI 0.5–12.3), the trend toward increased mortality in recurrent cases (7.0% vs. 2.7%) suggests heightened vulnerability due to more complex surgeries and a greater risk of complications. Further multicenter studies with larger sample sizes are needed to confirm this association.

Regarding LHS, patients with recurrence had an average stay 1 day longer, in line with literature showing prolonged hospitalization in these cases (16.4 vs. 9.5 days) [18]. Although this finding did not reach statistical significance in regression analyses, the trend supports the notion of increased postoperative complexity [31, 32]. Both models showed a low adjusted R^2^ and the presence of outliers, suggesting high individual variability and the possible influence of unmeasured clinical or socioeconomic factors on LHS.

This study has some limitations. The retrospective design carries an inherent risk of information bias; however, this was minimized through standardized data collection and training personnel. The patients with < 60 months of follow-up were excluded from the primary HCE group to ensure robust identification of non-recurrent cases. While this approach reduced the risk of misclassification, it may have underestimated outcomes, such as perioperative mortality and early postoperative complications in this group, potentially biasing comparisons between cohorts. Furthermore, the relatively low number of outcome events limited our ability to include multiple confounders in multivariable models and precluded adjusted analyses for mortality. Still, the use of robust regression techniques improved model reliability by controlling for overdispersion and outliers.

With regard to benzimidazole therapy, although it has been suggested to reduce recurrence, we were unable to evaluate this factor due to the lack of standardized data in our cohort. Current evidence on this issue remains of low certainty and derives mainly from small series and heterogeneous studies, as highlighted in international guidelines [6, 9, 17]. Regarding the classification of surgical procedures, it is important to acknowledge that terminology in the literature is not fully standardized. While some authors distinguish between radical and conservative procedures, others prefer the open- versus no-open-cyst classification [8, 24, 25]. To address this variability, we presented both approaches in our analysis. Nevertheless, direct comparisons with studies adopting different definitions should be interpreted with caution.

## Conclusion

This study demonstrates that the recurrence of HCE significantly increases the risk of POC. Although differences in mortality and LHS were not statistically significant, the observed trends suggest worse clinical outcomes in recurrent cases, potentially linked to surgical complexity and a higher complication burden. It is important to note that the independent effect of recurrence was evaluated after adjustment for evolutionary complications only, given the limited number of outcome events [33]. Further studies with larger cohorts are warranted to confirm this association while accounting for additional known prognostic factors.

These findings underscore the importance of long-term follow-up in patients undergoing surgery for HCE, with particular attention to recurrence prognostic factors [34]. Early identification of these predictors could help guide clinical decision-making and minimize postoperative morbidity. Moreover, recognizing recurrence as a key factor in surgical planning may enhance patient outcomes and optimize resource use in neglected tropical diseases.

## Data Availability

The data sets used are available from 10.5281/zenodo.16414121.
